# A Comparison and Analysis of Three Methods of Aluminum Crown Forgings in Processing Optimization

**DOI:** 10.3390/ma15238400

**Published:** 2022-11-25

**Authors:** Chi-Peng Chen, Hui-Zhen Su, Jyun-Kai Shih, Cheng-Fu Huang, Hao-Yun Ku, Chien-Wei Chan, Tomi-T. Li, Yiin-Kuen Fuh

**Affiliations:** 1 Department of Mechanical Engineering, National Central University, No. 300, Jhongda Rd., Jhongli Dist., Taoyuan 32001, Taiwan; 2 GLOBAL TEK., Xin-Wu Plant, No. 638, Sec. 6, Kuaisu Rd., XinWu Dist., Taoyuan 327, Taiwan

**Keywords:** response surface method (RSM), multiobjective optimization, main effects plot (MEP), grey relational analysis (GRA), controllable deformation zone (CDZ)

## Abstract

In this study, three parameter optimization methods and two designs of experiments (DOE) were used for the optimization of three major design parameters ((bill diameter (D), billet length (L), and barrier wall design (BWD)) in crown forging to improve the formability of aluminum workpiece for shock absorbers. The first optimization method is the response surface method (RSM) combined with Box–Behnken’s experimental design to establish fifteen (15) sets of parameter combinations for research. The second one is the main effects plot method (MEP). The third one is the multiobjective optimization method combined with Taguchi’s experimental design method, which designed nine (9) parameter combinations and conducted research and analysis through grey relational analysis (GRA). Initially, a new type of forging die and billet in the controlled deformation zone (CDZ) was established by CAD (computer-aided design) modeling and the finite element method (FEM) for model simulation. Then, this investigation showed that the optimal parameter conditions obtained by these three optimization approaches (RSM, MEP, and multiobjective optimization) are consistent, with the same results. The best optimization parameters are the dimension of the billet ((D: 40 mm, the length of the billet (L): 205 mm, and the design of the barrier wall (BWD): 22 mm)). The results indicate that the optimization methods used in this research all have a high degree of accuracy. According to the research results of grey relational analysis (GRA), the size of the barrier wall design (BWD) in the controllable deformation zone (CDZ) has the greatest influence on the improvement of the preforming die, indicating that it is an important factor to increase the filling rate of aluminum crown forgings. At the end, the optimized parameters are verified by FEM simulation analysis and actual production validation as well as grain streamline distribution, processing map, and microstructure analysis on crown forgings. The novelty of this work is that it provides a novel preforming die through the mutual verification of different optimization methods to solve a typical problem such as material underfill.

## 1. Introduction

Nowadays, forging technology has been widely used in different industry products [1]. Forging processing technology consists of squeezing material between a punch and a forging die to deform the material plastically to form a workpiece. According to the different recrystallization temperatures of the workpiece, it can be divided into cold working and hot working, as well as complex geometrical parts, which can be processed if the needs are required [2]. During the forging process, with the change in the processing temperature and the design of the product geometry, there are often problems with machining defects, resulting in poor product accuracy and poor formability. For example, folding defects are one of the most common defects, which can cause poor exterior quality, affecting the mechanical properties and material utilization of the final product [3,4]. Aluminum alloy 6066 constituted with Al-Mg-Si is used as a machined material in this study. The alloy is stronger and more brittle than ordinary aluminum alloys. Since 6066-T6 aluminum alloy contains Cu and Mn elements, the grain size can be prevented from becoming too large by adding the Mn element; furthermore, the precipitates can be refined, and the recrystallization temperature can increase the strength by containing Cu [5,6,7,8].

Moreover, during the forging process, the flow situation of the material also affects the filling effect and the forming force load of the forgings [9,10,11]. Forging technology involves a large number of experimental variables (for example, temperature, friction coefficient, and deformation rate), so the optimization process of the product requires a high experiment cost and long operation time. To reduce the investigation costs, improvements can be made by preforming the product and combining it with an FEM simulation to reduce the number of experiments [12,13,14]. Therefore, Jiang et al. [15] utilized FEM to simulate and study the causes of streamlined defects in the forming process of bearing rings to mend forging defects. Two hot forging methods were utilized to research the forging process, and the streamline distribution of the workpiece was improved. Chamanfar et al. [16] used a finite element (FE) model for the isothermal forging of nickel-based superalloys through model simulation. Relevant study data, including strain, temperature, and strain rate, were validated using grain size comparisons. The experimental outcomes prove that the FEM simulation results are consistent with the experimental data, indicating that the FEM helps to verify the rationality and accuracy of the research results. Equbal et al. [17] used FEM (DEFORM-3D) software to conduct finite element analysis of the hot forging process for spring saddles and used the Taguchi method to analyze the influence of forging the processing load and material temperature. It was found that billet temperature and flash thickness are the most important parameter factors. The forging process can achieve an improvement in lowering the billet temperature loss and reducing the forging load.

On the other hand, the response surface method (RSM) is an experimental design for optimizing forming processes. The number of experimental steps groups is lower, which can greatly save time and cost, and helps analyze optimized process parameters [18,19,20]. Recently, Meng et al. [21] used RSM merged with an FEM simulation to study the preforging of railway freight car couplers and proposed a closed-die forging method without flash, and also designed and optimized the preforming process of railway freight. The test results verified that the process was able to produce high-quality parts without forming defects. Qi et al. [22] established a parametric study of the forging process on large-diameter spur bevel gears through response surface analysis (RSA), took the forming load and die wear as the optimization goals, and then used FEM software (DEFORM-3D) to confirm the forming load and die wear in the traditional process. The Taguchi method was also utilized to plan the die forging experiments, and the wear test results were verified through physical and forging experiments after optimization analysis. 

For the study of the main effects plot method, Rasche et al. [23] studied low-energy flash-free crankshaft forging. The main effects plot method was used to confirm the effect of forming angle, cross-sectional area, and material temperature on workpiece flash. Silva et al. [24] conducted a friction stir welding (FSW) joint geometry optimization study for 6082 aluminum alloy, using the Taguchi method to design experiments with three different welded joints, as well as using the main effect plot to research the average effect on evaluating and optimizing the ultimate tensile strength (UTS) limit. Bansal et al. [25] investigated the metal removal rate (MRR) and surface roughness, and tested properties such as tensile strength and hardness, as well as the wear of two workpieces of alumina samples fabricated by sand casting. The main effects plot method was conducted for the study of the influence of process parameters such as depth of cut, machining speed, alumina particle concentration, and feed rate on surface roughness and MRR. 

The application aspect of multiobjective optimization methods is used in the fields of engineering, economics, and mathematics. It has high-efficiency global analysis capabilities for complex and nonlinear problems, is suitable for dealing with complex problems, and can effectively optimize process parameters [26,27,28]. Saedon et al. [29] adopted the Taguchi method as the experimental design method to study the effect of surface roughness, machining speed, and metal removal rate on wire electrical discharge machining (WEDM), and simplified the complex analysis process through the grey relation manner. In the end, the best parameters of the process were obtained. Younas et al. [30] studied the optimal processing parameters for Ti6Al4V alloy, optimized four experimental parameters—cutting energy, tool wear rate, surface roughness, and MRR—and compared them with multiobjective grey relational analysis. It was discovered that the feed rate and cutting speed are the parameters that have the greatest influence on the tool, and the optimized machining conditions have been confirmed by experiments to increase tool life and reduce surface roughness. Therefore, this study aims to develop various optimization methods combined with DOE to achieve optimal process parameters and to improve the formability of aluminum crown forgings for shock absorbers. 

## 2. Die Design of the Crown Forging

### 2.1. Original Die Design

**Figure 1:**
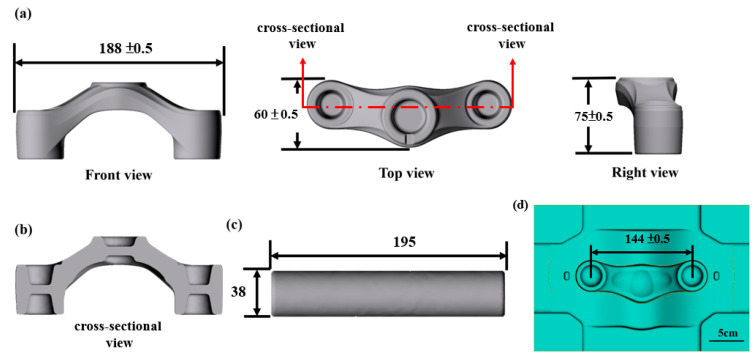
Original workpiece dimensions for crown forgings (in mm). (**a**) CAD model with views in front, top, and right; (**b**) a cross-sectional view of the crown forging workpiece; (**c**) relevant dimensions of the billet; (**d**) the dimension of bottom die.

AA6066-T6 crown forging is a critical part of the shock absorber of the bicycle. If the quality of the crown forging part is not good, the body structure may fail to pass the inspection. In the early stage of the study, to improve analysis efficiency and reduce research time, computer-aided design (CAD) technology was used to design and model forging dies according to the shapes and sizes of dies and preforming parts, as shown in Figure 1. The geometric dimensions of the length (188 ± 0.5 mm), width (60 ± 0.5 mm), and height (75 ± 0.5 mm) of the forging are shown in Figure 1a. Figure 1b explains the cross-sectional view of the internal shape design of the workpiece. Figure 1c is the CAD schematic diagram of the billet used in the forging of the workpiece. The diameter of the billet is 38 mm, and the length of the billet is 195 mm. The geometry characteristics of the forging die and the design of the bottom die are shown in Figure 1d. The workpiece is made of aluminum alloy 6066-T6. Table 1 lists the chemical composition of the material in this study.

**Figure 2:**
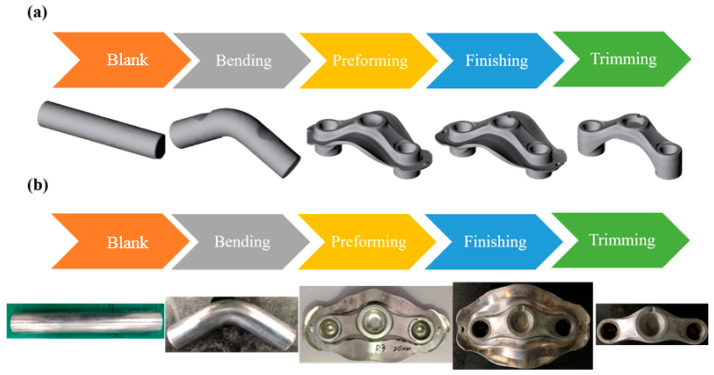
Process flow chart. (**a**) FEM model; (**b**) actual forging.

Figure 2a demonstrates the process steps of the forging workpiece in this study. First, a circular bar with a diameter of Ø38 mm is forged into a forging billet, and a preliminary volume distribution can be obtained at the same time. Second, the billet is bent into a preforming forging. The third step is to obtain the preforming parts of the crown forgings, and then complete all the forging processes by the crank-type mechanical forging press. After finishing the processed workpiece, the actual part (crown forging) is obtained after trimming, as shown in the actual forging flow chart of Figure 2b.

Table 2 lists the nomenclature and abbreviations used in this study, and Table 3 lists the FEM model simulation parameters for the forging process. A crank-type 10 MN mechanical forging press was modeled from the simulation software database. The flow stress of the billet was obtained using the database data of aluminum alloy 6066-T6 in QForm software. Due to the process requirements and equipment limitations of product developers, the temperature of the original billet was set to 480 °C and the die material was set to JIS SKD61. The temperature of the die was set at 130 °C, the constant shear friction between the forging die and the workpiece was 0.3, and the heat transfer coefficient (die to die) was set at 3000 W/m^2^ K.

### 2.2. Original Scheme Analysis of FEM for Model Simulation 

**Figure 3:**
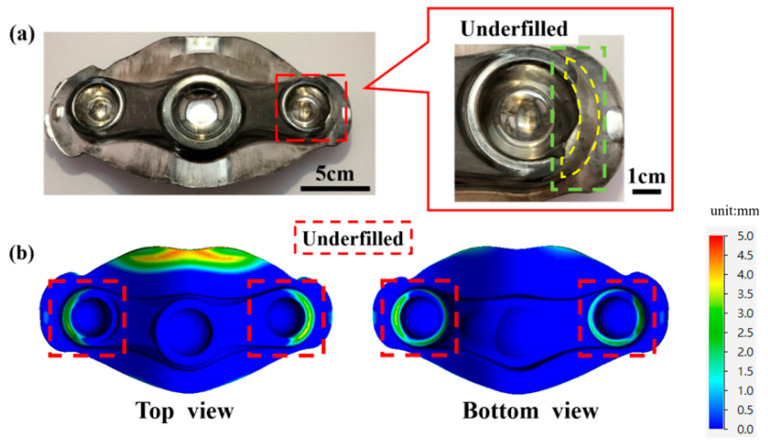
The crown original workpiece. (**a**) Defective forged crown products; (**b**) material-forging simulation diagram of defective crown products.

Figure 3a presents an incompletely filled workpiece using original process parameters: a billet diameter of 38 mm, a length of 195 mm, and a friction factor of 0.3 (frictional contact). The results of the QForm software (Version: 9.0.7) simulation show that the crowns on the left and right sides of the forging cannot be filled (defects), as shown in the green and yellow boxes in Figure 3a and the red box in Figure 3b. Figure 3b explains the material filling results from the simulation. Using CAD (computer-aided design) to model the crown forgings with model simulation by FEM, the same defect that the material cannot be formed completely is thus found in the crown workpiece. This FEM simulation proves that the design outcomes are consistent with the trend of the model in simulation results.

## 3. Optimization of the Preforming Die of Crown Forging

### 3.1. Experimental Design

**Figure 4:**
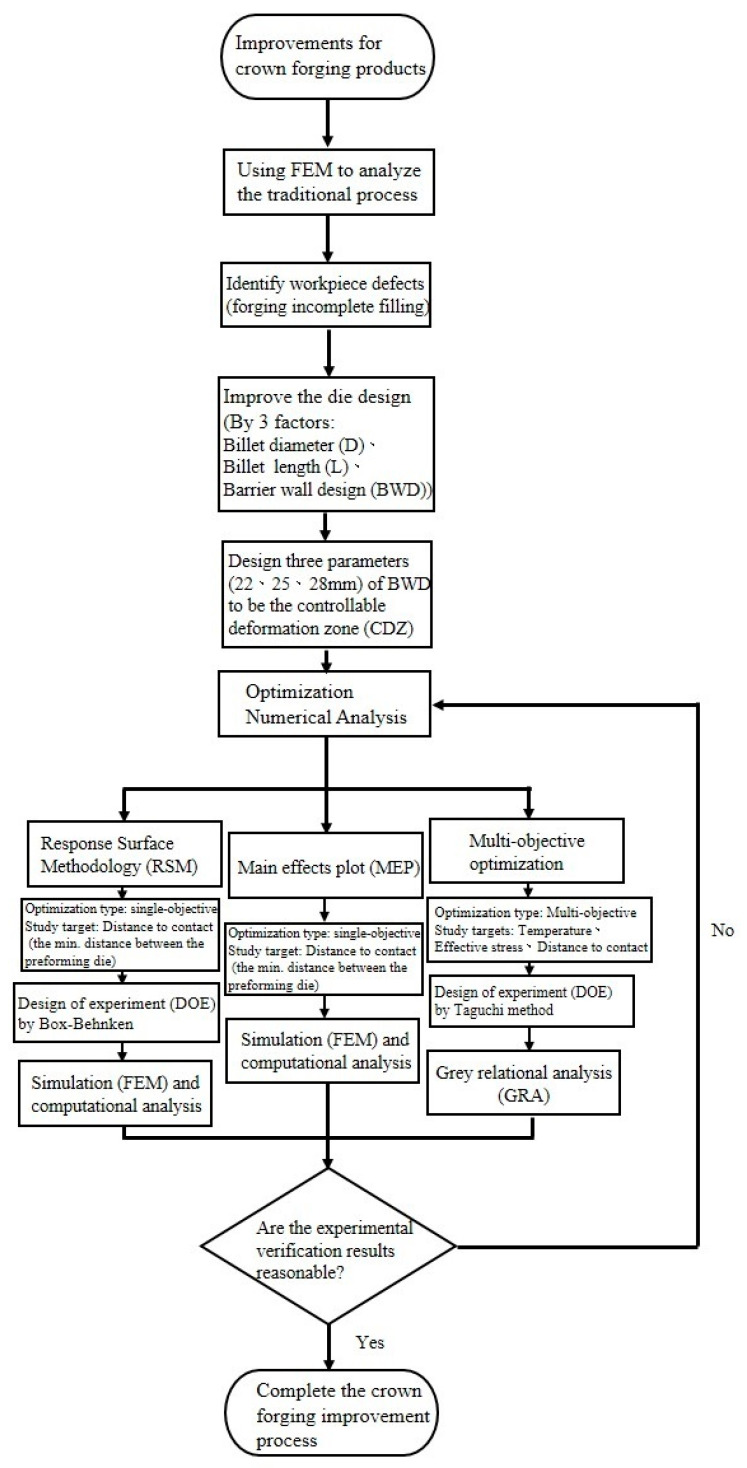
Flow chart of experimental design for workpiece optimization in this research.

Figure 4 explains the flow chart of the experimental design for crown forging optimization in this study. First, after determining the size of the workpiece and the manufacturing process as well as using FEM to analyze the traditional process, it was found that there was a problem of poor crown filling. Three research parameters ((billet diameter (D), billet length (L), and barrier wall design (BWD)) were used in preforming die design to improve the problem of material filling, where barrier wall design (BWD) was the controllable deformation zone (CDZ) of the die. Second, numerical analysis was performed using various optimization methods, including RSM, MEP, and multiobjective optimization methods to compare the results of these analyses with each other to discover the best machining parameters for the crown forging. Finally, the optimal process parameters were verified by simulation and experimental forming results, as well as microstructure analyses.

### 3.2. Design of Controllable Deformation Zone (CDZ)

**Figure 5:**
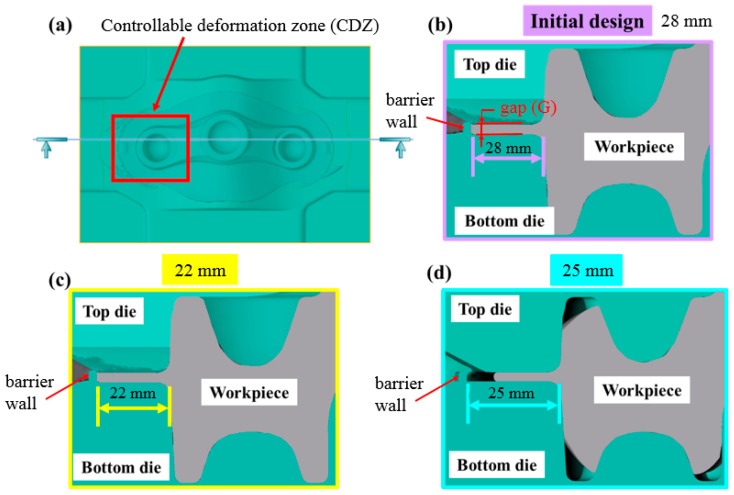
Optimization analysis of the preforming die, (**a**) A cross-sectional view of aluminum crown forging; (**b**) original BWD: 28 mm; (**c**) improved BWD: 22 mm; (**d**) improved BWD: 25 mm.

Figure 5 illustrates the optimization analysis process of the preforming die. Figure 5a is a schematic cross-sectional view of the die, and the controllable deformation zone is squarely framed in the crown area of the preforming die and is used to locate the problem of an incomplete forming place in the dimensional variation of the barrier wall. Figure 5b illustrates the FEA graph of the original design die; the design length of the original preforming die is 28 mm. Two new controllable deformation zone sizes are designed to enhance the accuracy and filling rate of crown forging. The design distances of the barrier wall are 22 mm and 25 mm, as shown in Figure 5c,d. Then, different analyses and verifications are carried out to find the best process parameters. The following study will describe the results for barrier wall design (BWD) variables.

### 3.3. Response Surface Methodology (RSM) Analysis

The RSM analysis method uses Box–Behnken design as a design of experiment, uses quadratic regression equations to fit the functional relationship between factors and response values, and then finds the optimal process parameters. Therefore, this investigation will combine practical production experience, mathematical methods, and finite element techniques to improve and optimize the design of the preforming die. The purpose of RSM analysis is to obtain the minimum gap (G), as shown in Figure 5b, between the top die and the bottom die after the crown forging forming process is completed. Utilizing the results of the variance analysis of the model determines the highest correlation factors with the gap (G). These correlation factors are billet diameter (D), the length of the billet (L), and the barrier wall design (BWD), listed in Table 4. Moreover, barrier wall design (BWD) is a very complex subject that needs to be conducted with a lot of experience. The barrier wall design (BWD) of the “barrier wall” proposed in this study is one of the main ways to increase the material filling rate. Other complex processes involving entire die refurbishment are not considered here in this study. 

To obtain the best target response results, 15 group parameter combinations were established by Box–Behnken design. Each scheme was simulated by the finite element method. The average statistics of the gap (G) between the top die and the bottom die are shown in Table 5. The parameter combination to obtain the minimum value of the gap (G) between the top die and the bottom die is D = 40 mm, L = 205 mm, and BWD = 22 mm.

According to the least-squares method and simulation results, the equation is obtained by RSM using the fitting function between three factors (D, L, and BWD) and the target (G). The gap equation between the top die and the bottom die:

G = 308.7 − 1.706 D − 2.694 L − 0.491 BWD + 0.01118 D^2^ + 0.00612 L^2^ + 0.00780 BWD + 0.00450 DL − 0.00525 DF + 0.00330 LF (1)

The variance analysis results of the gap (G) between the top die and the bottom die are shown in Table 6. Analysis of variance (ANOVA) is a common statistical analysis method for the collected data, the sum of square is a measure of variation from the mean, and the degrees of freedom are the number of independent pieces of information. Mean squares are the variance of the group data means, F-value is the ratio of two variances (mean square/mean-squared error), *p*-value is the probability, mean-squared error is the mean of the within group variances, and R is the correlation coefficient. Analysis of variance uses the sum of square and degree of freedom to estimate the mean of square of the parameter combination and to receive the final estimate of the *p* value. Based on the RSM principle of variance analysis, when the *p*-value is smaller than 0.05, it means that the influence of this factor is significant. When the *p*-value is smaller than 0.01, indicating that the prediction model is correct and effective. According to the results, the factors D, L, and BWD in the model related to the gap (G) have a significant impact. The correlation coefficient listed in Table 6 is 0.9968%, indicating that the gap prediction between the top die and the bottom die in the model is accurate, which proves that the analysis results are reasonable. Therefore, this method is suitable for subsequent parameter optimization.

**Figure 6:**
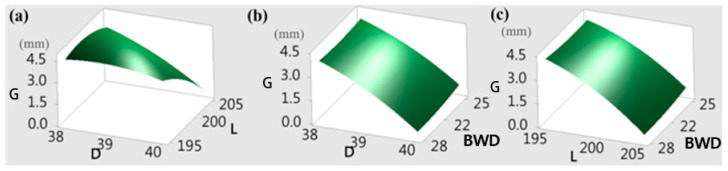
RSM analysis results for minimum gap (G) between the top die and the bottom die (**a**–**c**).

Figure 6 explains the results of a 3D response surface graph analysis of the gap (G) between the top die and the bottom die. Three factors (process parameters) are included in the response surface analysis: billet diameter, billet length, and barrier wall design (BWD) as shown in Figure 6a. According to the RSM results, when the combined parameters are D = 40 mm, L = 205 mm, and BWD = 22 mm, the minimum the gap (G) between the top die and the bottom die is 0 mm as shown in Figure 6b,c, which complies with the requirements of the dimensional accuracy of the workpiece. These outcomes are the same results as optimal values obtained by main effects plot method (MEP) and multiobjective optimization method (both are described below). 

### 3.4. Main Effects Plot (MEP) Analysis

The main effects plot (MEP) is a way to examine differences in means for different quantitative factor levels. The main effect occurs when different levels of a factor influence the response differently. This research needs further validation of the optimal parameter values for the workpiece by using main effects plots.

**Figure 7:**
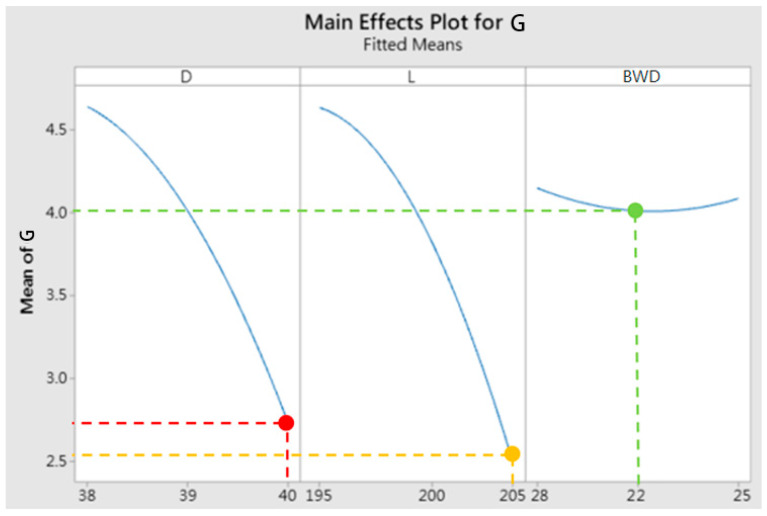
Main effects plot of the gap (G) between the top die and the bottom die.

Figure 7 illustrates the main effects plot (MEP) of three factors (billet diameter, billet length, and barrier wall design) of the gap between the top die and the bottom die. The vertical axis represents the mean G value in microns in the gap, and the horizontal axis is divided into three parts for each factor. The best value of the MEP for the D value is represented by the red line, the L value is represented by the yellow line, and the BWD value is represented by the green line. Because the purpose of this study is to increase the filling of the material, the minimum gap (G) value should be selected as the criteria of optimal process parameter. In the study of the main effects plot, the influence degree and trend of the three test factors on the gap were observed, and the size (diameter and length) of the billet has a greater influence on the gap (G) than the BWD factor; as shown in this figure, the gap range is 2.5~4.5 mm, and the larger the billet size, the smaller the gap (G) value, which means these two factors are in inverse ratio. This result indicates that the more material volume, the better the forging workability and the complete forming of the material. In addition, the smaller the BWD size, the smaller the gap (G) value, and the closer to the ideal target, this factor of BWD size is a direct ratio to the gap (G). This is because the size of the barrier wall is reduced, which can restrict the direction of the flow of material to help the material flow to the corner end of the die. This way can improve the formability of the workpiece. According to the analysis results, the optimized optimal parameters are D: 40 mm, L: 205 mm, and BWD: 22 mm. These results are the same as optimal values obtained by the response surface method (RSM) and multiobjective optimization method (as described below).

### 3.5. Multiobjective Optimization Analysis

Multiobjective optimization is a multicriteria decision-making method. Usually, multiple objectives are contradictory. To achieve the optimal value, some compromises must be made for multiple objectives. Therefore, this study uses the Taguchi method as a design of the experiment and utilizes the grey relational analysis method to obtain the influence degree of design parameters on crown forging quality for the shock absorber, and then obtains the optimal design parameter combination of the workpiece. 

Table 7 lists the resulting array of L_9_ (3^3^) orthogonal process parameters. The billet diameter ranges from 38 to 40 mm, the billet length ranges from 195 to 205 mm, and the barrier wall design (BWD) includes three design types (28 mm, 22 mm, and 25 mm).

Table 8 presents three different types of analytical values and signal-to-noise ratios used in this study, including temperature, effective stress, and gap, as temperature range: 482~496.5 °C, effective stress range: 40.27~57.08 MP, and gap range: 0.28~4.53 mm. The maximum S/N values for temperature, effective stress, and gap are -53.9184, −35.1297, and 11.0568, respectively. According to the results, the higher the signal-to-noise ratio is, the smaller the sensitivity of the crown forging quality that can be met due to the influence of the surrounding environment, which means that the item of high signal-to-noise ratio value is the best parameter for the forging process.

Multiobjective optimization was used in this study. The gamma correlation coefficients and SNR analysis values of the three factors (temperature, effective stress, and gap) are listed in Table 9. The analysis results of nine parameter combinations and their grey relational grade are illustrated in Table 10. Among them, the parameter combination of D: 40 mm, L: 205 mm, and BWD: 22 mm obtained the highest GRG correlation value: 0.7778. The greater the correlation of the parameter combination is, the better the parameters representing the combination can be set by the goals of the research.

**Figure 8:**
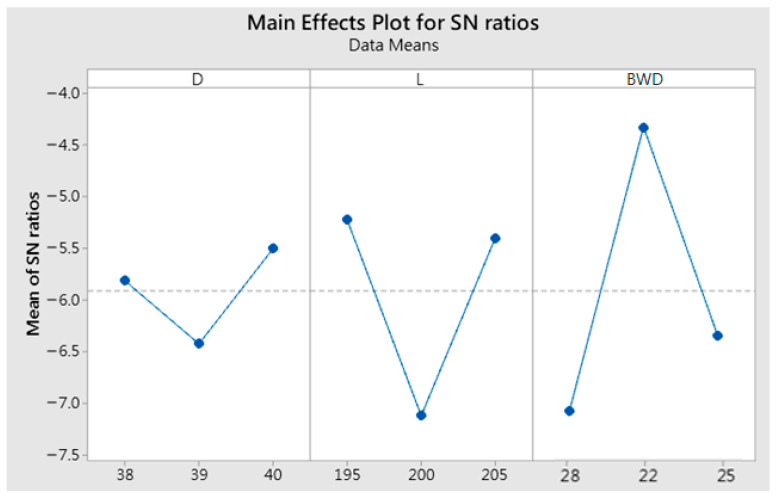
Response diagram for the grey relational grades.

The response analysis results according to the multiobjective gray correlation degree are shown in Figure 8. The influence of the barrier wall design (BWD) is the most significant, indicating that the design of the barrier wall will help to improve the accuracy and filling rate of the crown forging, while the diameter of the billet (D) has relatively little influence on the grey relational degree. 

In the multiobjective optimization method, various optimization combinations need to be determined, and the optimal parameters can only be determined after a comprehensive evaluation of multiple objectives. Among the optimal combination parameters, multiobjective optimization needs more consideration, because the most important factor in determining the optimization result is grey relational analysis. The experimental outcomes illustrated in Table 11 explain the outcomes after sorting by the grey relational grade. Therefore, the optimized parameters in this study are (D: 40 mm, L: 205 mm, BWD: 22 mm) design and the GRG value of this parameter combination is 0.778. These results are the same as the optimal values obtained by the response surface method (RSM) and main effects plot method (MEP).

## 4. Results and Discussion

The final goal of the workpiece is that the material must be filled in the die after forging. According to the previous research conclusions in this study, the parameters of the optimized process can be listed as follows: the diameter of the billet is 40 mm, the length of the billet is 205 mm, and the design length of the barrier wall design (BWD) is 22 mm.

**Figure 9:**
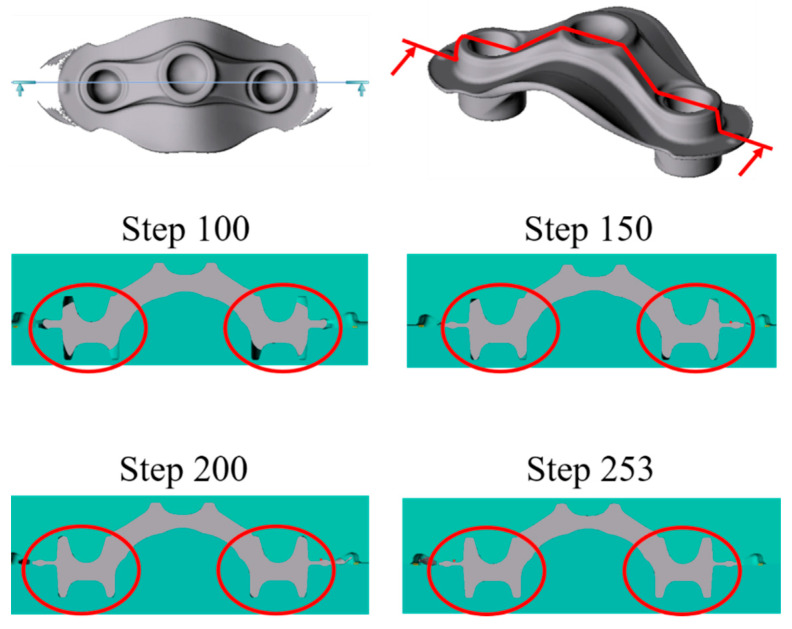
FEM simulation on forming and filling analysis of crown forgings.

The optimization parameters (D: 40 mm, L: 205 mm, and BWD: 22 mm) are used for FEM simulation. The number of simulated steps (100, 150, 200, and 253) is illustrated in Figure 9. The final simulation outcomes illustrate that the gap (G) between the top die and the bottom die is zero after the completion of 253 simulation steps, indicating that the crown forging is well filled, as marked by the red circle.

**Figure 10:**
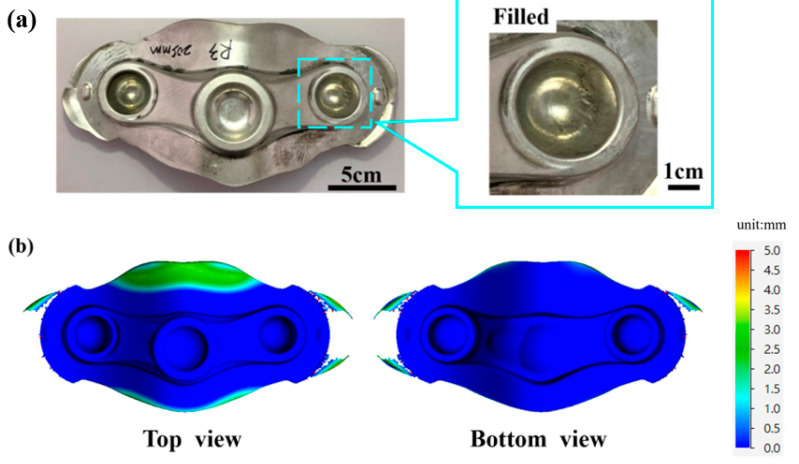
Improved crown-forging workpiece. (**a**) Top view of crown-forged products; (**b**) top view of simulation diagram of crown products.

Figure 10a illustrates the standard crown-forged product. The process parameters used are as follows: the diameter of the billet is 40 mm and the length is 205 mm, and the design length of the barrier wall design (BWD) is 22 mm. After processing, the crown of the workpiece is filled and has good formability (as shown in the blue box). Moreover, the simulation results explain that the crown can be filled as presented in Figure 10b. Therefore, it is proved that the optimized process parameter conditions obtained based on this study can improve the product-filling rate.

**Figure 11:**
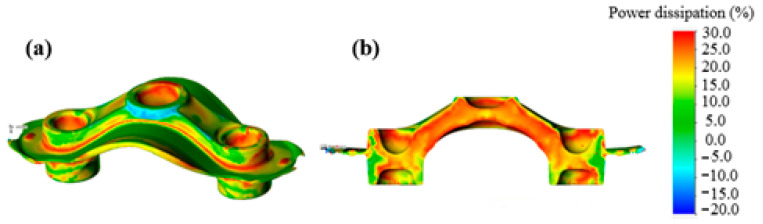
Processing map of AA6066-T6 workpiece. (**a**) Stereo view; (**b**) sectional view.

The simulation diagram of the AA6066-T6 workpieces is shown in Figure 11a. The power dissipation of the workpiece is uniformly distributed in the structure. The power dissipation value is not negative in the whole workpiece, indicating that there is no obvious processing instability area in the workpiece. The average power dissipation inside the workpiece is greater than 20% as shown in Figure 11b, which indicates that the workpiece forming is great, and the optimized process parameters in this study provide excellent workability.

**Figure 12:**
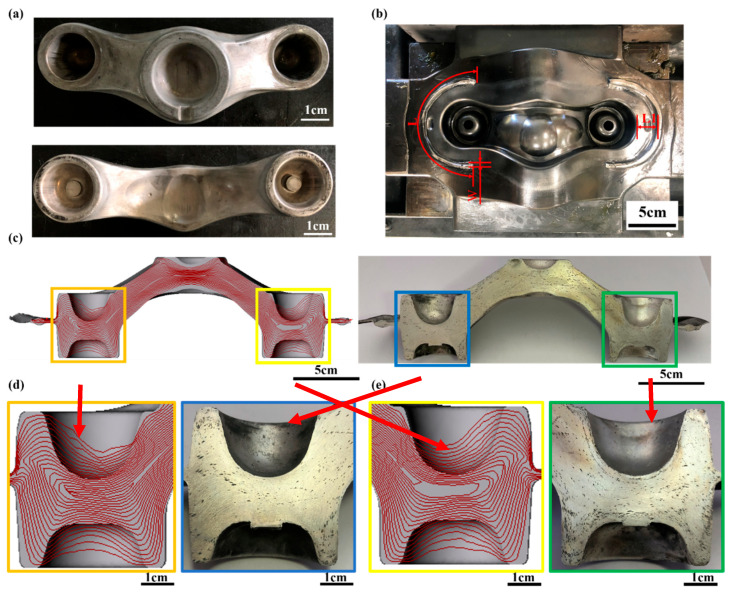
View of crown forgings. (**a**) Top and bottom view of the finishing forging; (**b**) view of actual counter (22 mm) with barrier wall design; (**c**) cross-sectional view of the finishing forging with grain streamlines (left: simulation; right: experiment); (**d**) enlarged view of the left side of the finishing forging with grain streamlines (orange squares: simulation; blue squares: experiment); (**e**) enlarged view of the right side of the finished forging with grain streamlines (yellow square: simulation; green square: experiment).

The top and bottom views of the finishing drawing of the crown forging are shown in Figure 12a. Figure 12b presents the design of the crown-forging die cavity of the bottom die, including the barrier wall (L = 142 mm, W = 2 mm) optimized with RSM to improve the filling efficiency of the forging and prevent excess metal from flowing out. Figure 12c explains the finite element simulation results (left) and experimental results (right) of the grain streamline distribution after the workpiece is forged. Figure 12d illustrates a partially enlarged view of the simulated (orange frame) and actual forging (blue frame) on the left side of the workpiece. Figure 12e indicates the simulated (yellow box) and actual forging (green box) on the right side of the workpiece. The partially enlarged view demonstrates the trend of the experimental and simulation results is consistent. The grain streamline distribution of the forging is dense and orderly, and the grain streamlines at the bottom of the groove gradually form a uniform streamline along the edge of the groove, which is consistent with the finite element simulation results (orange box). Therefore, according to the distribution of grain streamlines, the streamlines are smooth and do not intersect with dense streamlines, indicating that the workpiece is not prone to defects.

**Figure 13:**
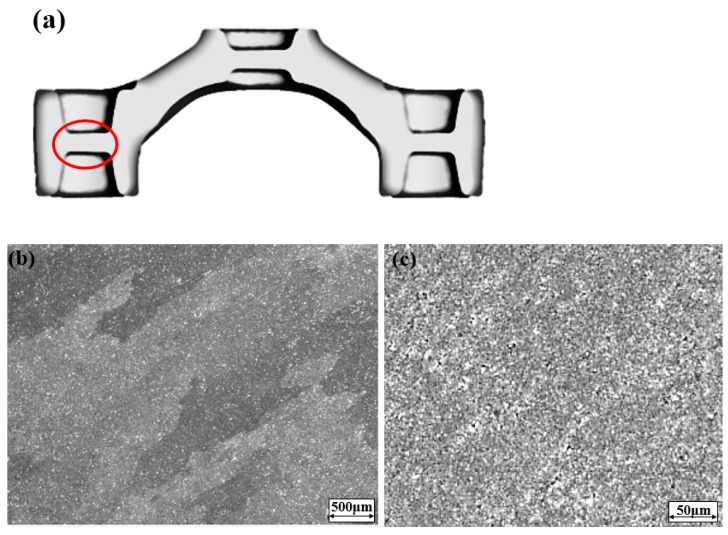
Microstructure of AA6066-T6 workpiece (true strain = 1). (**a**) Experimental observation position on the workpiece; (**b**) microstructure image of crown forgings (500 μm); (**c**) microstructure image of crown forgings (50 μm).

The metallographic examination is observed by the SEM (scanning electron microscope) instrument, and the observation position for the workpiece is shown in the red box of Figure 13a. The particle shape of the aluminum alloy workpiece after processing is roughly like a line shape, and the streamlined distribution of the particles becomes more evident with the improvement of the temperature of the deformation. In Figure 13b,c, there are many homogeneous microstructures, in which sizes are consistent and neatly arranged with no voids on the workpiece. The average particle size is small, and average grain radius is 2.65 microns. These results indicate that the process parameters chosen in this study can make the deformation of microstructure of the workpiece very uniform, with excellent forming properties [31,32].

## 5. Conclusions

Traditional manufacturing procedures usually use a single-objective optimization method that leads to the inaccuracy of the experimental results and causes the problem of insufficient formability of crown forgings. The main contribution of this study achieves 100% formability of crown forgings for the shock absorber, mainly because different analytical methods were studied on the deformation mechanism of the workpiece to obtain the optimization parameters. After the processing of thousands of pieces of this improved crown forging, the product yield rate exceeds 90%. This study provides a new preforming die design to improve crown forging formability. According to the results, the following conclusions are drawn:

1. According to the analysis of the RSM method, when the factor value (*p* value) in the model is smaller than 0.05, it means that the influence of the factor is significant. From the RSM analysis results, it is known that the factors of D, L, and BWD are most closely related to the gap (G) between the top die and the bottom die. The correlation coefficient is as high as 0.9968%.
2. According to the main effects plot (MEP) analysis, the size (diameter and length) of the billet has a greater influence on the gap (G) than the BWD factor, and the larger the billet size, the smaller the gap (G) value, which means that these two factors are in inverse ratio. This result explains that the more material volume, the better the forging workability and the complete forming of the material. In addition, the smaller the BWD size, the smaller the gap (G) value; this factor of BWD size is a direct ratio to the gap (G). This is because the size of the barrier wall is reduced, which can restrict the direction of the flow of material to help the material flow to the corner end of the die. This way can improve the formability of the workpiece.
3. Based on the outcomes of the response analysis from the multiobjective gray correlation degree, the influence of barrier wall design (BWD) is the most significant. It indicates that the creation of the partition wall is of great help to improve the accuracy and filling rate of the product, while the blank diameter (D) has the most minor effect in the comparison of the gray correlation degree. To analyze the process parameters of the numerical performance of the gamma correlation coefficient and signal-to-noise ratio of each combination for three objectives (temperature, effective stress, and minimum gap), the experimental results explain that the optimized parameters are as follows: D—40 mm, L—205 mm, and BWD—22 mm. These results are the same as optimal values obtained by the response surface method (RSM) and main effects plot method (MEP).
4. The power dissipation value of the crown forging inside the processing map reaches an average of 20%, and there is almost no negative value, which means the workpiece workability is great. On the other hand, the metallographic analysis demonstrates that the crown forging has a uniform grain distribution and almost no voids with small average particle sizes. These results prove that the optimized parameter combinations and preforming designs for this workpiece are the best choice in these analyses.

## Figures and Tables

**Table 1:** Chemical composition of the material.

Elements	Al	Mg	Si	Cu	Mn	Fe	Cr	Zn
AA6066-T6 Minimum–Maximum content (wt. %)	>95.86	0.8–1.4	0.9–1.8	0.7–1.2	0.6–1.1	<0.50	<0.4	<0.25

**Table 2:** Nomenclature and abbreviations used in this study.

Nomenclature	Abbreviation
ANOVA	Analysis of variance
BWD	Barrier wall design
CAD	Computer-aided design
CDZ	Controlled deformation zone
DOE	Designs of experiments
FEM	Finite element method
GRA	Grey relational analysis
GRG	Grey relational grade
MEP	Main effects plot method
RSM	Response surface method
SNR	Signal-to-noise

**Table 3:** The parameters of the forging process of the crown forgings.

Parameters	Unite	Value
Blank temperature	°C	480
Die temperature	°C	130
Material of blank	/	AISI Aluminum alloy 6066-T6
Material of die	/	JIS SKD61
Heat transfer coefficient (blank to die)	kw/m^2^ °C	3
Friction factor	/	0.3
Velocity of top die	Stroke/min	90
Element style	/	Tetrahedron
Mesh size of blank	mm	1
Mesh size of dies	mm	1–16
Mesh number		100,000–200,000
Friction coefficient		0.3
Heat transfer coefficient	W/m^2^ K	3000

**Table 4:** The variable value range of preforming die.

Variable	Unit	Lower Line	Median Line	Top Line
Diameter (D)	mm	38	39	40
Length (L)	mm	195	200	205
Barrier wall design (BWD)	mm	22	25	28

**Table 5:** Experimental and simulation results of various parameter combinations.

Scheme	Factor 1 Billet Diameter (D) mm	Factor 2 Billet Length (L) mm	Factor 3 Barrier Wall Design (BWD) mm	Target Gap (between the Top Die and the Bottom Die) (G) mm
Contribution (%)	34.84	55.02	10.02	
1	39	205	25	2.57
2	39	205	25	3.05
3	38	200	22	4.23
4	39	200	28	3.73
5	38	205	22	3.96
6	40	200	22	2.71
7	40	200	28	2.82
8	40	195	28	4.10
9	39	195	28	4.48
10	39	195	22	4.60
11	40	205	22	0
12	39	200	22	3.90
13	39	200	22	3.83
14	38	195	25	4.92
15	38	200	25	4.65

**Table 6:** Variance analysis of the gap (G) between the top die and the bottom die.

Factor	Sum of Square	Degree of Freedom	Mean Squares	F-Value	*p*-Value
Model	0.031963	9	0.003551	479.42	<0.001
D	0.029561	1	0.029561	3990.50	<0.001
L	0.001447	1	0.001447	195.36	<0.001
BWD	0.000514	1	0.000514	69.33	<0.001
DL	0.000020	1	0.000020	2.73	0.159
DF	0.000028	1	0.000028	3.72	0.112
LF	0.000044	1	0.000044	5.88	0.060
D2	0.000029	1	0.000029	3.90	0.105
L2	0.000138	1	0.000138	18.7	0.008
F2	0.000224	1	0.000224	30.29	0.003
Mean Squared error	0.000037	5	0.000007		
Correlation coefficient		R2 = 0.9968			

**Table 7:** L_9_ (3^3^) orthogonal array of process parameters.

No.	Blank Diameter [mm] D	Blank Length [mm] L	Barrier Wall Design [mm] BWD
1	38	195	28
2	39	200	22
3	40	205	25
4	39	195	22
5	39	200	25
6	39	205	28
7	40	195	25
8	40	200	28
9	40	205	22

**Table 8:** Analysis values and S/N ratios.

No.	Temperature		Effective Stress		Gap (G)	
	Analysis Value [°C]	S/N (dB)	Analysis Value [MPa]	S/N (dB)	Analysis Value [mm]	S/N (dB)
1	482.1	−53.6627	40.27	−32.0996	4.53	−13.1220
2	482.6	−53.6717	43.24	−32.7177	4.02	−12.0845
3	482.7	−53.6735	43.8	−32.8295	3.61	−11.1501
4	483.8	−53.6933	47.65	−33.5613	4.32	−12.7097
5	483.4	−53.6861	44.73	−33.0120	3.14	−9.9386
6	483.6	−53.6897	46.15	−33.2834	2.07	−6.3194
7	483.8	−53.6933	45.52	−33.1640	3.89	−11.7990
8	484	−53.6969	40.86	−32.2260	1.47	−3.3463
9	496.5	−53.9184	57.08	−35.1297	0.28	11.0568

**Table 9:** Grey relational coefficients and S/N ratios for the three quality characteristics.

No.	Temperature		Effective Stress		Gap (G)	
	S/N (dB)	Grey Relational Coefficient	S/N (dB)	Grey Relational Coefficient	S/N (dB)	Grey Relational Coefficient
1	−53.6717	0.3412322	−32.0996	0.3333333	−13.1220	1
2	−53.6627	0.3333333	−32.7177	0.3778377	−12.0845	0.8064516
3	−53.6735	0.3428571	−32.8295	0.3875951	−11.1501	0.6978654
4	−53.6933	0.361809	−33.5613	0.4712644	−12.7097	0.9100642
5	−53.6861	0.3546798	−33.0120	0.4049627	−9.9386	0.6045519
6	−53.6897	0.358209	−33.2834	0.4347039	−6.3194	0.4634678
7	−53.6933	0.361809	−33.1640	0.4209867	−11.7990	0.7685353
8	−53.6969	0.3654822	−32.2260	0.3413198	−3.3463	0.4098361
9	−53.9184	1	−35.1297	1	11.0568	0.3333333

**Table 10:** Nine (9) experimental groups and GRG (grey relational grade).

No.	Blank Diameter [mm] D	Blank Length [mm] L	Barrier Wall Design [mm] BWD	Grey Relational Grade
1	38	195	28	0.55818852
2	38	200	22	0.505874221
3	38	205	25	0.476105869
4	39	195	22	0.581045884
5	39	200	25	0.454731461
6	39	205	28	0.418793563
7	40	195	25	0.517110345
8	40	200	28	0.372212699
9	40	205	22	0.777777778

**Table 11:** Grey relational analysis for multiobjective optimization.

No.	D (mm)	L (mm)	BWD (mm)	Grey Relational Grade of Temperature	Grey Relational Grade of Effective Stress	Grey Relational Grade of Gap (G)	Grey Relational Grade	Rank
1	38	195	28	0.341	0.333	1	0.558	3
2	38	200	22	0.333	0.377	0.806	0.506	5
3	38	205	25	0.343	0.387	0.698	0.476	6
4	39	195	22	0.362	0.471	0.910	0.581	2
5	39	200	25	0.355	0.405	0.605	0.455	7
6	39	205	28	0.358	0.435	0.463	0.419	8
7	40	195	25	0.362	0.421	0.769	0.517	4
8	40	200	28	0.365	0.341	0.410	0.372	9
9	40	205	22	1	1	0.333	0.778	1
Optimal	40	205	22	1	1	0.333	0.778	

## Data Availability

The authors confirm that the data supporting the findings of this study are available within the article.

## References

[1] Hawryluk M., Ziemba J., Sadowski P. (2017). A Review of Current and New Measurement Techniques Used in Hot Die Forging Processes. Meas. Control.

[2] Hawryluk M. (2016). Review of selected methods of increasing the life of forging tools in hot die forging processes. Arch. Civ. Mech. Eng..

[3] Gao P., Yan X., Fei M., Zhan M., Li Y. (2019). Formation mechanisms and rules of typical types of folding defects during die forging. Int. J. Adv. Manuf. Technol..

[4] Skryabina N., Aptukov V., Rango P.D., Fruchart D. (2020). Effect of temperature on fast forging process of Mg-Ni samples for fast formation of Mg_2_Ni for hydrogen storage. Int. J. Hydrog. Energy.

[5] Zhen L., Fei W.D., Kang S.B., Kim H.W. (1997). Precipitation behaviour of Al-Mg-Si alloys with high silicon content. J. Mater. Sci..

[6] Hatch J. (1984). Aluminum: Properties and Physical Metallurgy.

[7] Mondolfo L. (1976). Aluminum Alloys Structure & Properties.

[8] Lin C., Hung F., Lui T., Chen L. (2016). High-temperature deformation resistance and forming behavior of two-step SIMA-processed 6066 alloy. Mater. Sci. Eng. A.

[9] Sun W., Chen L., Zhang T., Zhang K., Zhao G., Wang G. (2018). Preform optimization and microstructure analysis on hot precision forging process of a half axle flange. Int. J. Adv. Manuf. Technol..

[10] Su K., Welo T., Wang J. (2018). Improving friction drilling and joining through controlled material flow. Procedia Manuf..

[11] Lee S., Jo J., Joun M., Lee J. (2019). Efect of friction conditions on material flow in FE analysis of Al piston forging process. Int. J. Precis. Eng. Manuf..

[12] Kampen D., Richter J., Blohm T., Knust J., Langner J., Stonis M., Behrens B. (2020). Design of a genetic algorithm to preform optimization for hot forging processes. Int. J. Mater. Form..

[13] Nytra M., Kubík P., Petruška J., Šebek F. (2020). A Fully Coupled Thermomechanical damage analysis of hot closed die forging using finite element modeling. J. Mater. Eng. Perform..

[14] Kumar S.D., Purushothaman K., Chandramohan D., Dushyantraj M.M., Sathish T. (2020). ANN-AGCS for the prediction of temperature distribution and required energy in hot forging process using finite element analysis. Mater. Today Proc..

[15] Jiang H., Wu Y., Gong X., Shan D., Zong Y. (2020). Control of flow lines during the forging process of bearing outer rings with a deviated groove. Int. J. Adv. Manuf. Technol..

[16] Chamanfar A., Valberg H.S., Templinc B., Plumeri J.E., Misiolek W.Z. (2019). Development and validation of a finite-element model for isothermal forging of a nickel-base superalloy. Materialia.

[17] Equbal M.I., Kumar R., Shamim M., Ohdar R.K. (2014). A grey-based Taguchi method to optimize hot forging process. Procedia Manuf. Sci..

[18] Zhang L., Yue Y. (2018). Influence of waste glass powder usage on the properties of alkali-activated slag mortars based on response surface methodology. Constr. Build. Mater..

[19] Xie W., Jiang W., Wu Y., Song H., Deng S., Lăzărescu L., Zhang S., Banabic D. (2022). Process parameter optimization for thin-walled tube push-bending using response surface methodology. Int. J. Adv. Manuf. Technol..

[20] Honarpisheh M., Jobedar M.M., Alinaghian I. (2018). Multi-response optimization on single-point incremental forming of hyperbolic shape Al-1050/Cu bimetal using response surface methodology. Int. J. Adv. Manuf. Technol..

[21] Meng F., Cai Z., Chen Q. (2019). Multi-objective optimization of preforming operation in near-net shape forming of complex forging. Int. J. Adv. Manuf. Technol..

[22] Qi Z., Wang X., Chen W. (2019). A new forming method of straight bevel gear using a specific die with a flash. Int. J. Adv. Manuf. Technol..

[23] Rasche N., Langner J., Stonis M., Behrens B. (2018). Experimental investigation of different parameters at a combined cross wedge rolling and multi-directional forging process. Prod. Eng..

[24] Silva A.C.F., Braga D.F.O., Figueiredo M.A.V., Moreira P.M.G.P. (2015). Ultimate tensile strength optimization of different FSW aluminum alloy joints. Int. J. Adv. Manuf. Technol..

[25] Bansal P., Upadhyay L. (2016). Effect of Turning Parameters on Tool wear, surface roughness and metal removal rate of alumina reinforced aluminum composite. Procedia Technol..

[26] Kitayama S., Kadoya S., Takano M., Kobayashi A. (2021). Multi-objective optimization of process parameters in cold forging minimizing risk of crack and forging energy. Arch. Civ. Mech. Eng..

[27] Lv N., Liu D., Hu Y., Yang Y., Wang J. (2022). Multi-objective optimization of parametric design for profile ring rolling process based on residual stress control. Int. J. Adv. Manuf. Technol..

[28] Prakash J.U., Ananth S., Sivakumar G., Moorthy T.V. (2018). Multi-Objective Optimization of Wear Parameters for Aluminum Matrix Composites (413/B4C) using grey relational analysis. Mater. Today Proc..

[29] Saedona J.B., Jaafar N., Yahaya M.A., Saada N.H., Kasim M.S. (2014). Multi-objective optimization of titanium alloy through orthogonal array and grey relational analysis in WEDM. Procedia Technol..

[30] Younas M., Jaffery S.H.I., Khan M., Khan M.A., Ahmad R., Mubashar A., Ali L. (2019). Multi-objective optimization for sustainable turning Ti6Al4V alloy using grey relational analysis (GRA) based on analytic hierarchy process (AHP). Int. J. Adv. Manuf. Technol..

[31] Ravichandran N., Prasad Y.V.R.K. (1991). Dynamic recrystallization during hot deformation of aluminum: A study using processing maps. Metall. Mater. A.

[32] Zhang C., Wang C., Guo R., Zhao G., Chen L., Sun W., Wang X. (2019). Investigation of dynamic recrystallization and modeling of microstructure evolution of an Al-Mg-Si aluminum alloy during high-temperature deformation. J. Alloys Compd..

